# Comment on Calugi et al. The Role of Weight Suppression in Intensive Enhanced Cognitive Behavioral Therapy for Adolescents with Anorexia Nervosa: A Longitudinal Study. *Int. J. Environ. Res. Public Health* 2023, *20*, 3221

**DOI:** 10.3390/ijerph20176690

**Published:** 2023-08-31

**Authors:** Adrian Meule

**Affiliations:** 1 Department of Psychiatry and Psychotherapy, LMU University Hospital, LMU Munich, Nußbaumstr. 7, 80336 Munich, Germany; ameule@med.lmu.de; 2 Schoen Clinic Roseneck, Am Roseneck 6, 83209 Prien am Chiemsee, Germany

Calugi and colleagues [1] recently reported on a study that examined the effects of weight suppression on treatment outcomes in adolescents with anorexia nervosa. Weight suppression refers to the difference between a person’s current and highest body weight at their current height [2]. There is a plethora of studies showing that higher weight suppression is a predictor of larger weight gain in non-clinical samples and in samples with people with eating disorders [3,4,5,6]. As the authors investigated adolescents, they used developmental weight suppression and standardized body mass index (z-BMI) in their analyses, but for readability, I will just use the terms weight suppression and BMI when referring to these variables.

Amongst other analyses, the authors computed linear regression analyses using BMI at the end of treatment and at follow-up as dependent variables. They state in the statistical analyses section that “confounding variables included in the model were: age, illness duration, and z-BMI, EDE-Q, BSI, and CIA global scores recorded at baseline” (p. 4). However, they then state in the results section that BMI at baseline “was excluded because of the high multicollinearity” (p. 6). However, this is crucial, as the authors report that weight suppression was negatively related to BMI at the end of treatment and at follow-up and state in the discussion section that this finding is in contrast to findings from other studies. They then also speculate about possible reasons for this, such as different samples studied in terms of age.

However, the coefficients for weight suppression in the regression models (reported in Table S1 in the Supplementary Material of the article [1]) merely quantify the between-subjects association between weight suppression and BMI at the end of treatment and at follow-up, respectively, when holding age, illness duration, and questionnaire scores at baseline constant. That is, they do not represent any information related to changes in BMI. Weight suppression is usually uncorrelated or weakly negatively correlated to (current) BMI, and this is what these coefficients replicate.

To examine whether weight suppression predicts weight change, BMI at baseline needs to be included as an independent variable in the regression models [7]. The authors should not worry about multicollinearity when coefficients in a regression model that include multiple predictors are properly interpreted [8]. Similarly, the selection of covariates should be based on conceptual considerations, not on statistical criteria [9]. Furthermore, it has been suggested that coefficients in models that include covariates should always be reported with and without the covariates so it can be seen what difference adding the covariates makes [10].

Thus, I suggest that the authors reanalyze their data by running models that only include weight suppression and BMI at baseline as independent variables when predicting BMI at the end of treatment and at follow-up (and maybe additionally examine models with the other covariates included in a second step). When controlling for BMI at baseline, the coefficient for weight suppression then represents the association between weight suppression and BMI change. Thus, I hypothesize that the coefficient for weight suppression will now be positive, indicating that higher weight suppression at baseline relates to larger weight gain, in line with findings from the extant literature.
